# Predictors and pathways of in-hospital mortality in active vascular contrast extravasation detected on abdominopelvic CT

**DOI:** 10.1186/s13244-024-01748-y

**Published:** 2024-07-12

**Authors:** Rathachai Kaewlai, Gun Chomchalerm, Sasima Tongsai, Jitti Chatpuwaphat, Anchisa Chatkaewpaisal, Pramuk Khamman, Somrach Thamtorawat, Banjerd Praditsuktavorn, Worapat Maitriwong, Junichi Matsumoto

**Affiliations:** 1 Department of Radiology, Faculty of Medicine Siriraj Hospital, Mahidol University, 2 Wanglang Rd, Bangkok Noi, Bangkok, 10700 Thailand; 2 Department of Research, Faculty of Medicine Siriraj Hospital, Mahidol University, 2 Wanglang Rd, Bangkok Noi, Bangkok, 10700 Thailand; 3 Department of Anatomy, Faculty of Medicine Siriraj Hospital, Mahidol University, 2 Wanglang Rd, Bangkok Noi, Bangkok, 10700 Thailand; 4 Department of Surgery, Faculty of Medicine Siriraj Hospital, Mahidol University, 2 Wanglang Rd, Bangkok Noi, Bangkok, 10700 Thailand; 5 Department of Emergency and Critical Care Medicine, St. Marianna University School of Medicine, Kawasaki, Kanagawa Japan

**Keywords:** Contrast extravasation (blood), Tomography (X-ray computed), Hemorrhage, Mortality, Adults

## Abstract

**Objectives:**

This study aimed to identify factors influencing in-hospital mortality in adult patients with active vascular contrast extravasation (AVCE) on abdominopelvic computed tomography (CT).

**Methods:**

All consecutive patients with AVCE detected on CT between January 2019 and May 2022 were retrospectively included. Their data were compared through uni- and multivariable analyses between patients with and without in-hospital mortality. Path analysis was utilized to clarify the relationships among factors affecting mortality.

**Results:**

There were 272 patients (60.2 ± 19.4 years, 150 men) included, of whom 70 experienced in-hospital mortality. Multivariable analysis revealed nonsurgery, chronic kidney disease (CKD) stage 4–5 or dialysis, prolonged partial thromboplastin time (PTT), minimum AVCE length > 8 mm, and a lower rate of packed red cell (PRC) transfusion were identified as independent predictors of in-hospital mortality (*p* = 0.005–0.048). Path analysis demonstrated direct influences of CKD4-5 or dialysis, prolonged PTT, and minimum AVCE length on mortality (coefficients 0.525–0.616; *p* = 0.009 to < 0.001). PRC transfusion impacted mortality through nonsurgery (coefficient 0.798, *p* = 0.003) and intensive care unit (ICU) admission (coefficients 0.025, *p* = 0.016), leading to subsequent death. Three AVCE spaces (free, loose, and tight) defined on CT were not directly associated with in-hospital mortality.

**Conclusion:**

In adults with AVCE on CT, AVCE size had a direct independent influence on mortality, highlighting the critical role of radiologists in detecting and characterizing this finding. Additionally, CKD4-5 or dialysis and prolonged PTT also directly influenced mortality, while the lower rate of PRC transfusion impacted mortality through nonsurgery and ICU admission.

**Clinical relevance statement:**

In patients with active vascular contrast extravasation (AVCE) on abdominopelvic CT, larger AVCE directly increased in-hospital mortality. Radiologists’ detection and characterization of this finding is crucial, along with recognizing factors like CKD4-5, dialysis, and prolonged PTT to improve patient outcomes.

**Key Points:**

- Several factors independently predicted in-hospital mortality in patients with abdominopelvic AVCE.
- Extravasation length > 8 mm was the only imaging marker predictive of in-hospital mortality.
- Non-imaging factors correlated with in-hospital mortality, and PRC transfusion impacted mortality through nonsurgery and ICU admission pathways.

**Graphical Abstract:**

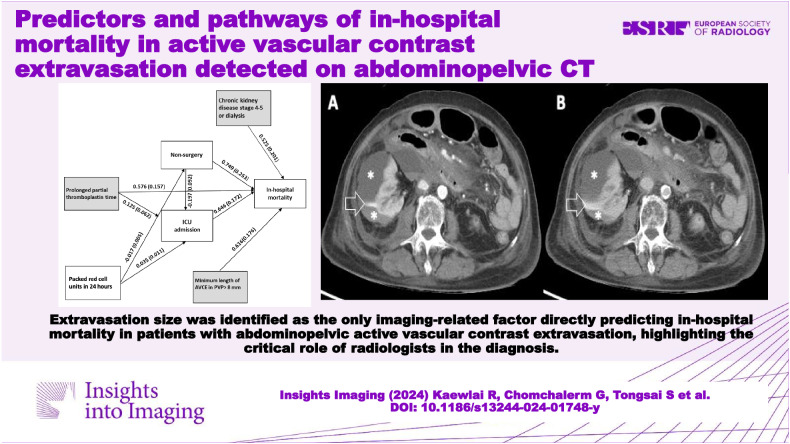

## Introduction

Active vascular contrast extravasation (AVCE) is a critical CT finding, often requiring emergent treatment. First reported in 1989, AVCE manifests as a focal collection of contrast material adjacent to injured organs with high-attenuated hemoperitoneum [1]. Subsequent investigations consistently underscore the importance of AVCE and the reliability of CT in diagnosing and localizing AVCE [2–5]. The benefits of CT include rapid acquisition, noninvasiveness, and precise localization of bleeding sites, aiding in treatment selection and procedural planning. Given the prominence of nonsurgical interventions like transarterial embolization (TAE) and endoscopy in managing abdominopelvic bleeding [2, 4–6], understanding prognostic factors is imperative. Our retrospective study aimed to identify clinical and CT-based factors associated with in-hospital mortality in adults with AVCE on abdominopelvic CT. Recognizing these factors informs acute management decisions, potentially reducing morbidity and mortality. By elucidating the pathways to death in patients with AVCE, valuable insights into the complexity of bleeding-related deaths are gained. Healthcare providers can anticipate and manage potential complications, tailoring treatment strategies effectively. These pathways may inform clinical decision-making and resource allocation, thereby optimizing patient care.

## Methods

### Study design and patient selection

**Fig. 1:**
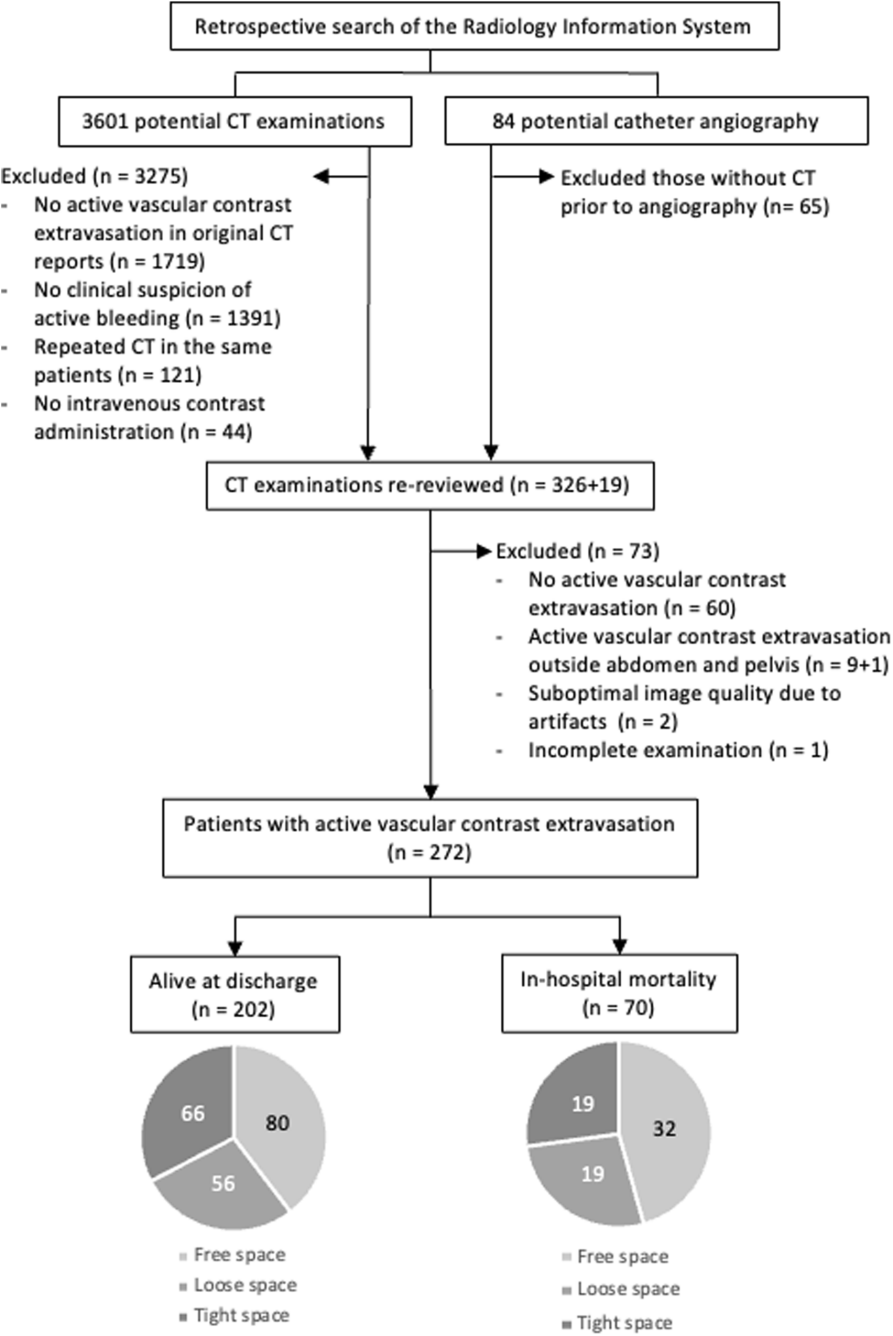
Flowchart of patient inclusion

The retrospective study, approved by our hospital’s Institutional Review Board (approval number Si 822/2023), was conducted at a 2200-bed academic hospital. The informed consent was waived due to the retrospective nature. Figure 1 shows the patient inclusion flow chart. We searched the hospital’s Radiology Information System (RIS) database for abdominopelvic CT and catheter angiography reports between January 2019 and May 2022, identifying 575 reports of adult patients (age ≥ 18 years) containing keywords related to active bleeding with images available in Picture Archiving and Communication System (PACS). After re-review by an emergency radiologist (R.K.) with 20 years of experience, 272 CT examinations with AVCE were included. A pilot study with 41 participants (26.8% mortality rate) informed our threshold determination (95% confidence level; maximum error margin of 20% relative to the identified mortality rate translating to an absolute error of 5.4%), requiring at least 263 CT scans to estimate in-hospital mortality accurately. The patient clinical information, laboratory results, treatment, and outcome data were collected by searching the hospital’s electronic database.

### Clinical data abstraction

The clinical information was abstracted from electronic medical records by two physicians (A.C. and P.K.), who were knowledgeable about patients suspected active bleeding history. While aware of the research objectives, they remained blinded to specific AVCE locations. Data included demographics, physical examinations, laboratory results, diagnoses, durations, treatments, CT, and angiographic findings.

### Image acquisition

The CT examinations obtained at our institution (*n* = 242) were conducted using one of four multidetector scanners (Revolution CT, GE Healthcare), with axial images reconstructed at a 1.25-mm slice thickness. Parameters included 120 kVp, 250 mAs, and a pitch ratio of 0.99. At least one postcontrast phase (arterial phase; AP, portovenous phase; PVP, delayed phase; DP) was performed, covering the upper border of the hemidiaphragms to the ischial tuberosities. Timing for AP, PVP, and DP was set at 35–45 s, 70–80 s, and 5–10 min or more postcontrast administration, respectively. CT examinations from outside hospitals (*n* = 30) had a maximum slice thickness of 2 mm and at least one postcontrast phase available. All images were stored in our PACS (Synapse, Fujifilm Corporation).

### Image reinterpretation and definitions of spaces of active vascular contrast extravasation

All 272 CT scans were independently re-reviewed for AVCE spaces by two radiologists (J.C. and R.K.) specialized in emergency and body imaging with 7 and 20 years of experience who were blinded to patient history details, clinical data, and diagnosis except for patient age, sex, and clinical presentation suspicious of active bleeding. Each reviewer assigned a space for each AVCE (Table 1), and all disagreements were resolved by a third radiologist (J.M.) who specialized in emergency imaging and intervention with 22 years of experience. Reports of angiography with or without embolization were reviewed for details, and any results inconsistent with CT findings received a re-review by an interventional radiologist (S.T.).

**Table 1:** Comparison of patient and treatment characteristics between patients with active vascular contrast extravasation who remained alive at discharge and those who died in the hospital (*n* = 272)

Factors	All patients (*n* = 272)	Alive at discharge (*n* = 202)	In-hospital mortality (*n* = 70)	*p*-value*
**Mean age (years; SD)**	60.2 (19.4)	58.0 (19.2)	66.6 (18.4)	0.001
**Male sex (*n*, %)**	150 (55.1)	120 (59.4)	30 (42.9)	0.024
Chronic kidney disease stage 4–5 or dialysis	37 (13.6)	21 (10.4)	16 (22.9)	0.016
**Nontraumatic etiologies**	205 (75.4)	142 (70.3)	63 (90.0)	0.002
**Vital signs** (mean, SD)				
Systolic blood pressure (mmHg)	122.4 (23.6)	124.5 (23.6)	116.4 (22.7)	0.013
Diastolic blood pressure (mmHg)	71.9 (16.5)	73.4 (15.7)	67.7 (18.1)	0.012
Pulse pressure (mmHg)	50.5 (18.5)	51.1 (18.1)	48.7 (19.6)	0.355
Pulse rate (beats/min)	95.6 (22.1)	95.1 (21.3)	97.0 (24.3)	0.520
**Laboratory results** (mean, SD)				
Hemoglobin (g/dL; *n* = 254)	7.9 (3.1–38.0)	8.6 (3.1–38.0)	7.3 (3.5–11.7)	≤ 0.001
Hematocrit (%; *n* = 255)	24.0 (9.4–51.5)	25.3 (9.4–51.5)	21.7 (11.6–34.5)	< 0.001
Platelet (× 10^3^ cell/mm^3^, *n* = 254)	166.5 (5.0–1003.0)	198.0 (5.0–1003.0)	122.0 (34.0–393.0)	< 0.001
Prothrombin time (sec) (*n* = 243)	14.8 (10.2–64.2)	14.4 (10.2-64.2)	17.9 (11.9-47.2)	< 0.001
Partial thromboplastin time (sec) (*n* = 243)	27.9 (12.0-144.0)	27.1 (18.0-81.0)	32.2 (12.0-144.0)	< 0.001
International normalized ratio (*n* = 178)	1.4 (1.0-9.0)	1.3 (1.0–7.0)	1.7 (1.0-9.0)	< 0.001
Base excess (*n* = 87)	−6.1 (−26.0–14.0)	−5.4 (−21.0–14.0)	−7.6 (−26.0–8.0)	0.169
Lactate (mg/dL) (*n* = 146)	4.3 (1.0–24.0)	3.7 (1.0–24.0)	6.3 (1.0–24.0)	0.001
**Packed red cells** (*n* = 269)				
Use (n, %)	222 (82.5)	156 (78.0)	66 (95.7)	0.002
Units in 24 h (median, min-max)	3.0 (0–21.0)	2.0 (0–17.0)	4.0 (0–21.0)	< 0.001
**Time intervals** (hours; median, min-max)				
From CT to angiography (*n* = 169)	5.3 (0.4–164.7)	5.3 (0.4–164.7)	5.1 (0.6–148.3)	0.136
From CT to surgery or other procedures (*n* = 64)	16.4 (0.2–312.6)	15.8 (0.2–201.7)	16.4 (0.4–312.6)	0.659
From CT to any treatment (*n* = 206)	5.3 (0.2–201.7)	5.3 (0.2–201.7)	5.5 (0.4–139.4)	0.314
**Angiography**				
Any (*n*, %)	171 (62.9)	130 (64.4)	41 (58.6)	0.472
With embolization (*n*, %)	145/168 (86.3)	107/128 (83.6)	38/40 (95.0)	0.110
**Surgery** (*n*, %; *n* = 270)	49 (18.1)	43 (21.4)	6 (8.7)	0.029
**Pharmacological adjuncts** (*n*, %; *n* = 270)	104 (38.5)	70 (34.8)	34 (49.3)	0.047
**Other procedures** (*n*, %; *n* = 270)	37 (13.7)	28 (13.9)	9 (13.0)	1.000
**None of above treatments** (n, %; *n* = 270)	31 (11.5)	22 (10.9)	9 (13.0)	0.800
**ICU admission** (*n,* %)	155 (57.0)	100 (49.5)	55 (78.6)	< 0.001
**Length of stay** (days; median, min-max)				
ICU	1.5 (0–170.0)	0 (0–107)	13 (0–170)	< 0.001
Total	16.0 (0–355.0)	14.0 (0–355.0)	28.0 (0–170.0)	0.002
**Timing of death from index CT** (*n*, %; *n* = 70)	N/A	N/A		N/A
< 24 h			7 (10.0)	
> 24 h to 7 days			17 (24.3)	
> 7 days			46 (65.7)	
**Follow-up CT**				
Use (*n*, %)	128 (47.1)	96 (47.5)	32 (45.7)	0.902
Number of studies (median, min-max)	0 (0–7)	0 (0–7)	0 (0–6)	0.682
**Rebleeding** (*n*, %; *n* = 270)	27/270 (10.0)	16/200 (8.0)	11/70 (15.7)	0.105

*CT* computed tomography, *ICU* intensive care unit

* Values underlined represent statistical significance

### Counts and CT characteristics of active vascular contrast extravasation

**Fig. 2:**
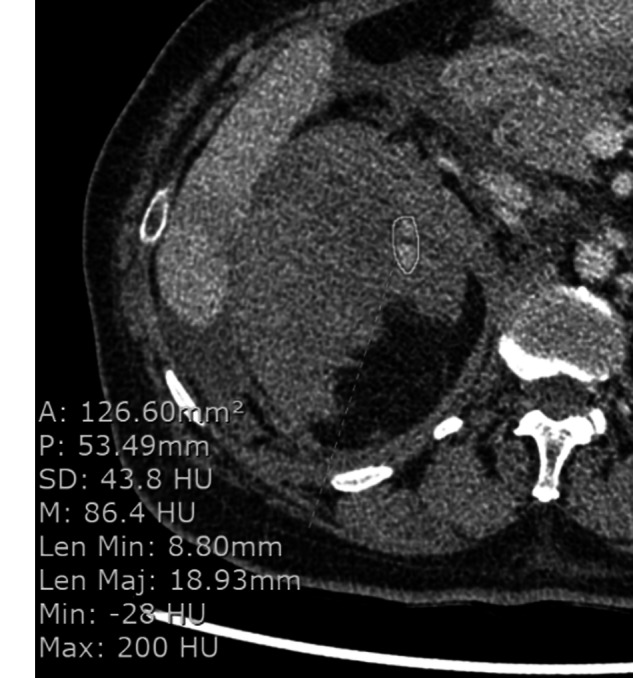
Example of measurements of active vascular contrast extravasation on a CT image using electronic calipers. A area (mm^2^), Len Max maximum length (mm), Len Min minimum length (mm), M mean (Hounsfield units; HU), Max maximum (HU), Min minimum (HU), P perimeter (mm), SD standard deviation (HU)

The “representative” AVCE in this investigation was the only one that a patient had in the CT examination. If there were more than one AVCEs in the same organ or structure, the largest one was selected as a representative of the examination. If there were more than one site of AVCE in different organs or structures, the one in a space easier for the spread of AVCE was chosen (i.e., the free-space AVCE selected over loose- or tight-space ones). All measurements were made on a standard PACS workstation as shown in Fig. 2 with an electronic caliper for obtaining the size and CT attenuation of AVCE. These values were recorded in terms of area (mm^2^), perimeter (mm), minimal diameter (mm), maximal diameter (mm), mean attenuation (Hounsfield units; HU), and standard deviation (SD) of attenuation (HU). These parameters were calculated as percentages of changes between two pairs of CT phases: AP vs. PVP, AP vs. DP, and PVP vs. DP.

### Reference standards

The diagnosis of AVCE was made using standard CT criteria. AVCE was defined as a focal, diffuse, or jet-like collection outside a vessel with attenuation close to that of the aorta or major adjacent arteries, and higher than surrounding parenchymal organ. In multiphase CT, AVCE can change or merge into an enhanced hematoma in later images [6, 7]. AVCE spaces were assigned according to its location and propensity to spread, detailed in Table E1.

### Statistical analysis—general, uni- and multivariable analyses

Categorical attributes such as sex, underlying diseases, medications, clinical history (traumatic or nontraumatic origin), laboratory results (grouped), and AVCE spaces were represented as numbers or percentages. Continuous data, such as age, length of hospitalization, and time until treatment, were presented as either mean with SD for normally distributed data or median with range for skewed data.

To investigate potential factors associated with mortality during the same hospital admission, comparisons were made using Pearson chi-square, Yates’ continuity correction, or Fisher’s exact test for categorical data, and independent t-test or Mann-Whitney U test for normal continuous data or skewed data, respectively. Univariable and multivariable binary logistic regression analyses were conducted to identify factors linked to in-hospital mortality. The effect size and direction were quantified using odds ratios (OR) with 95% confidence intervals (CI), using Statistical Package for Social Sciences for Windows (SPSS, version 27, IBM). A significance level of 0.05 was adopted.

Following comprehensive multivariable logistic regression analysis, we further explored the interrelations of variables and effects on in-hospital mortality using path analysis. Path analysis, a component of structural equation modeling (SEM), dissects the complex interplay between identified independent variables and multiple dependent variables, including mediators and outcome variables. It models both direct and indirect relationships within a set of variables, providing a robust framework for elucidating the underlying mechanisms governing the observed associations. This approach allowed us to simultaneously consider multiple dependent variables, accommodating the complexity of our conceptual model. It helped uncover nuanced pathways linked the variables of interest. The path model was constructed step by step, starting with the initial model, which included all paths originating from the independent variables and traversing through potential mediators before culminating in the outcome. Subsequent models were formulated by systematically excluding specific paths from the initial model, aiming to refine the model while retaining its explanatory power.

Fit indices, including the Comparative Fit Index (CFI), Tucker-Lewis Index (TLI), Root Mean Square Error of Approximation (RMSEA), and Standardized Root Mean Square Residual (SRMR), were computed for all path models. Good fit was indicated by RMSEA and SRMR ≤ 0.08, and CFI and TLI > 0.95. The final model was selected for its less complexity but statistically significant path coefficients for all included paths. The lavaan package in R version 4.3.2 was employed for initial model construction, using structural equation modeling (SEM).

## Results

There were 272 adult patients (150 men, mean age of 60.2 ± 19.4 years) with 272 CT examinations. AVCE was nontraumatic in 205 and traumatic in 67 cases. Most patients had AVCE in one organ/structure (255/272; 93.8%) with the most frequent being gastrointestinal (GI) tract (62/272; 22.8%), retroperitoneum (46/272; 16.9%), intramuscular (42/272; 15.4%), and peritoneal cavity (28/272; 10.3%). 222 patients (222/272; 81.6%) received packed red cells (PRC) transfusion with a median of 3 units (range; 0–21) in the first 24 h of active bleeding. TAE was performed in 145 patients (145/272; 53.3%), while pharmacologic adjuncts, surgical hemostasis, and other procedures such as endoscopic hemostasis were performed in 104 (104/272; 38.2%), 49 (49/272; 18.0%), and 37 patients (37/272; 13.6%), respectively. 155 patients (155/272; 57.0%) were admitted in the ICU. The median length of stays in the hospital and ICU of were 16 days (range; 0–355), and 1.5 days (range; 0–170), respectively. Rebleeding occurred in 27 patients (27/270; 10%). Seventy patients (70/272; 25.7%) died in the same admission while the rest were alive at the time of discharge. Tables 1 and 2 provided overall patient information and compared characteristics (patient, CT, and treatment-related) between those who were alive at discharge and those with in-hospital mortality.

**Table 2:** Comparison of computed tomography characteristics between patients with active vascular contrast extravasation who remained alive at discharge and those who died in the hospital (*n* = 272)

Factors	All patients (*n* = 272)	Alive at discharge (*n* = 202)	In-hospital mortality (*n* = 70)	*p*-value*
**Time from event to CT** (hours; median, min-max)	5.1 (0.1–96.0)	5.0 (0.1–96.0)	5.9 (0.5–62.3)	0.330
**Numbers of organ with AVCE** (*n,* %)				0.322
1	255 (93.8)	189 (93.6)	66 (94.3)	
2	15 (5.5)	12 (5.9)	3 (4.3)	
3	1 (0.4)	1 (0.5)	0 (0)	
4	1 (0.4)	0 (0)	1 (1.4)	
**Organ of AVCE**				0.461
* Gastrointestinal tract*	*62 (22.8)*	*41 (20.3)*	*21 (30.0)*	0.133
* Peritoneal cavity*	*37 (13.6)*	*28 (13.9)*	*9 (12.9)*	0.993
* Mesentery*	*6 (2.2)*	*6 (3.0)*	*0*	0.344
* Post-surgical space*	*6 (2.2)*	*4 (2.0)*	*2 (2.9)*	0.649
* Renal pelvocaliceal system*	*1 (0.4)*	*1 (0.5)*	*0*	1.000
Renal angiomyolipoma	1 (0.4)	1 (0.5)	0	1.000
Pelvic extraperitoneal space	13 (4.8)	12 (5.9)	1 (1.4)	0.194
Retroperitoneal space	46 (16.9)	32 (15.8)	14 (20)	0.539
Spleen	2 (0.8)	2 (1.0)	0	1.000
Subcutaneous fat	13 (4.8)	9 (4.5)	4 (5.7)	0.746
* Adrenal glands*	*2 (0.8)*	*1 (0.5)*	*1 (1.4)*	0.449
* Intramuscular*	*42 (15.4)*	*30 (14.9)*	*12 (17.1)*	0.791
* Intratumoral*	*1 (0.4)*	*1 (0.5)*	*0*	1.000
* Renal parenchyma*	*6 (2.2)*	*4 (2.0)*	*2 (2.9)*	0.649
* Liver*	*14 (5.1)*	*13 (6.4)*	*1 (1.4)*	0.125
* Ovary*	*1 (0.4)*	*1 (0.5)*	*0*	1.000
* Pancreas*	*1 (0.4)*	*1 (0.5)*	*0*	1.000
* Rectus sheath, abdominal wall musculature*	*17 (6.3)*	*15 (7.4)*	*2 (2.9)*	0.253
* Uterus*	*1 (0.4)*	*0*	*1 (1.4)*	0.257
**Number of AVCE in organ of interest**				
Median, min-max	1 (1–10)	1 (1–10)	1 (1–5)	0.720
≥ 2 sites (*n*, %)	55 (20.4)	42 (20.8)	13 (18.6)	0.863
**AVCE first shown in the arterial phase** (*n*, %)	235 (86.4)	176 (87.1)	59 (84.3)	0.692
**Space of AVCE** (*n,* %)				0.611
Free	112 (41.2)	80 (39.6)	32 (45.7)	
Loose	75 (27.6)	56 (27.7)	19 (27.1)	
Tight	85 (31.3)	66 (32.7)	19 (27.1)	
**Angiographically positive AVCE** (*n*, %) (*n* = 168)	90/168 (53.6)	65/126 (51.6)	25/42 (59.5)	0.475
**Area of AVCE** (mm^2^; median, min-max)				
AP (*n* = 229)	47.9 (1.3–571.2)	47.3 (1.3–571.2)	49.3 (4.2–392.6)	0.850
PVP (*n* = 264)	116.2 (2.5–967.0)	106.3 (2.5–936.7)	142.9 (22.9–967.0)	0.051
Delayed phase (*n* = 174)	197.2 (13.2–2792.7)	187.3 (13.2–2792.7)	229.1 (48.3–1788.6)	0.373
**Perimeter of AVCE** (mm; median, min-max)				
AP (*n* = 229)	39.6 (10.0–192.1)	38.7 (10.0–192.1)	40.9 (10.2–142.7)	0.809
PVP	59.4 (11.1–312.9)	56.9 (11.1–312.9)	66.0 (21.6–248.0)	0.162
Delayed phase (*n* = 174)	80.1 (15.5–352.6)	76.6 (15.5–352.6)	83.0 (33.0–305.9)	0.945
**Minimum length of AVCE** (mm; median, min-max)				
AP (*n* = 229)	6.7 (0.7–39.9)	6.8 (0.7–39.9)	6.4 (2.0–22.1)	0.960
PVP (*n* = 264)	10.2 (1.1–45.2)	9.6 (1.1–45.2)	12.2 (4.6–39.4)	0.028
Delayed phase (*n* = 174)	13.8 (3.8–61.9)	13.4 (3.8–58.4)	15.1 (6.5–61.9)	0.492
**Maximum length of AVCE** (mm; median, min-max)				
AP (*n* = 228)	13.3 (3.2–58.0)	13.3 (3.2–58.0)	14.4 (3.3–45.2)	0.926
PVP (*n* = 264)	20.0 (3.5–81.4)	19.5 (3.5–81.4)	22.1 (6.9–70.8)	0.236
Delayed phase (*n* = 174)	28.2 (5.0–107.5)	27.9 (5.0–101.4)	28.4 (10.9–107.5)	0.814
**Mean attenuation of AVCE** (HU; median, min-max)				
AP (HU; *n* = 229)	128.3 (60.5–480.4)	124.3 (60.5–347.7)	135.8 (77.6–480.4)	0.211
PVP (HU) (*n* = 264)	124.0 (52.8–441.5)	121.7 (52.8–313.6)	130.0 (59.5–441.5)	0.127
Delayed phase (HU; *n* = 174)	94.1 (53.2–189.0)	92.5 (53.2–189.0)	100.4 (55.0–169.9)	0.207
**Standard deviation of attenuation of AVCE** (HU; median, min-max)				
AP (HU; *n* = 208)	54.4 (19.5–182.1)	53.7 (19.5–182.1)	55.8 (27.2–100.8)	0.572
PVP (HU; *n* = 259)	50.0 (16.1–252.6)	47.3 (16.1–167.0)	53.0 (22.7–252.6)	0.059
Delayed phase (HU; *n* = 174)	35.2 (13.9–87.4)	34.3 (13.9–71.8)	36.7 (22.6–87.4)	0.023

*AP* arterial phase, *AVCE* active vascular contrast extravasation, *CT* computed tomography, *HU* Hounsfield Unit, *PVP* portovenous phase

* Values underlined represent statistical significance

### In-hospital mortality of patients with AVCE

Bleeding in free, loose, and tight spaces accounted for 45.7%, 27.1%, and 27.1% of all mortality. When evaluating AVCE on an organ/structure basis, the highest mortality rate was among GI AVCE (30%), followed by retroperitoneal AVCE (20%) and intramuscular AVCE (17.1%).

### Comparison between patients without and with in-hospital mortality (Tables 1 and 2)

In-hospital mortality was identified more frequently in older female patients with a greater number of underlying diseases, low blood pressures, hemoglobin, hematocrit, platelets, and prolonged prothrombin time, partial thromboplastin time (PTT), and international normalized ratio (*p* < 0.05). They were often treated in an ICU for more days, received PRC transfusion in a greater quantity, had pharmacological adjunctive treatment, and did not undergo surgery (Table 1). On CT (Table 2), their AVCEs had a larger area (*p* = 0.051), and minimum diameter (*p* = 0.028) on the PVP. They appeared more heterogeneous in attenuation—manifested as SD of AVCE—when viewed in the DP CT, and this heterogeneity was most pronounced between the AP and other phases (*p* = 0.016–0.039). Multivariable analysis (Table 3) revealed five factors independently associated with in-hospital mortality, which included nonsurgery, chronic kidney disease (CKD) stage 4–5 or dialysis, prolonged PTT, minimum AVCE length in PVP ≥ 8 mm, and higher rate of PRC transfusion. A detailed evaluation of CT findings suggested that AVCE heterogeneity (Table E2) manifested as SD of CT attenuation of AVCE in DP, differences of these values between AP and PVP, and AP and DP were dependent to in-hospital mortality with odds ratios ranging from 1.007 to 8.075 and areas under the receiver operating characteristics (ROC) curve (AUC) between 0.611 and 0.615.

**Table 3:** Uni- and multivariable analyses of predictive factors of in-hospital mortality in patients with active vascular contrast extravasation

Factors	Univariable model		Multivariable model^a^	
	Unadjusted OR (95%CI)	*p*-value	Adjusted OR (95% CI)	*p*-value
**Chronic kidney disease stage 4-5 or dialysis**	2.554 (1.246, 5.236)	0.010	5.622 (1.402, 22.539)	0.015
**Prolonged partial thromboplastin time**	2.741 (1.543, 4.867)	< 0.001	4.568 (1.565, 13.337)	0.005
**Packed red cell units in 24** **h**	1.113 (1.035, 1.196)	0.004	1.181 (1.001, 1.394)	0.048
**Nonsurgery**	2.858 (1.159, 7.048)	0.023	31.409 (2.151, 458.171)	0.012
**Minimum length of AVCE in PVP** > **8** **mm**	2.349 (1.239, 4.455)	0.009	3.965 (1.043, 15.070)	0.043

*AVCE* active vascular contrast extravasation, *CI* confidence interval, *OR* odds ratio, *PVP* portovenous phase

^a^ *Analysis adjusted for all factors* found to be associated with same-admission mortality in the *univariable analysis* (*p* < 0.05), i.e. age, sex, chronic kidney disease stage 4–5 or dialysis, any 1 of 3 underlying diseases, DBP < 60 mmHg, hemoglobin, hematocrit, prothrombin time, partial thromboplastin time, partial thromboplastin time < 31 sec, international normalized ratio, PRC use, PRC units in 24 h, surgery, pharmacological adjuncts, ICU admission, ICU length of stay, and total length of stay

### Path analysis and SEM

**Fig. 3:**
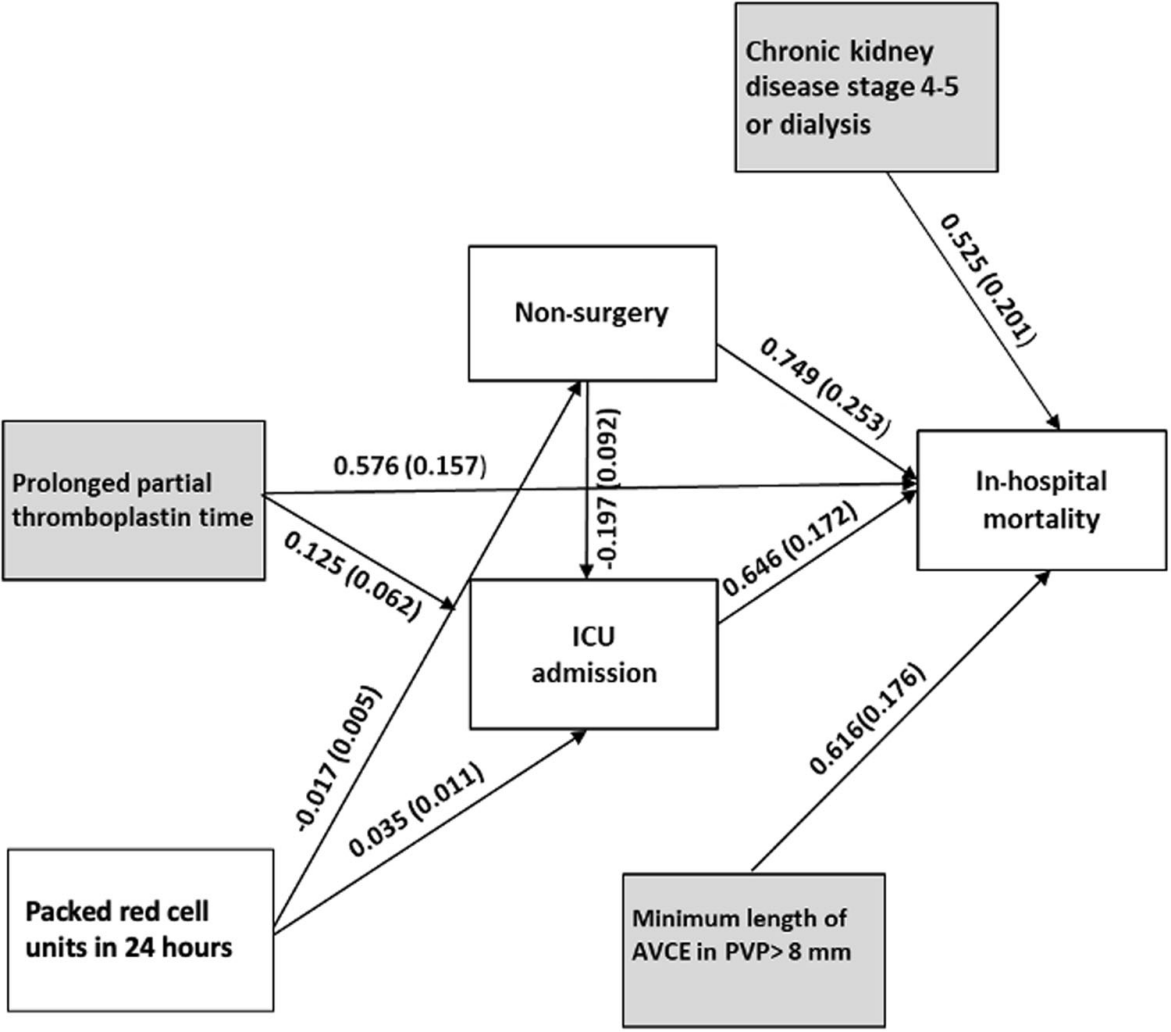
Path analysis demonstrates relationships among factors leading to in-hospital mortality in patients with active vascular contrast extravasation detected on abdominopelvic CT. The values were standardized parameter estimates with standard errors in brackets. Values on solid lines indicated statistical significance (*p*-value < 0.05), while those on dashed lines were not statistically significant. Items in grey boxes directly influenced the outcome (in-hospital mortality), while those in white boxes either indirectly affected (PRC) or acted as mediators (Nonsurgery, and ICU) toward the outcome. CKD4-5, chronic kidney disease stage 4–5; ICU, intensive care unit; PRC, packed red cell; PTT, partial thromboplastin time; PVP, portovenous phase

**Fig. 4:**
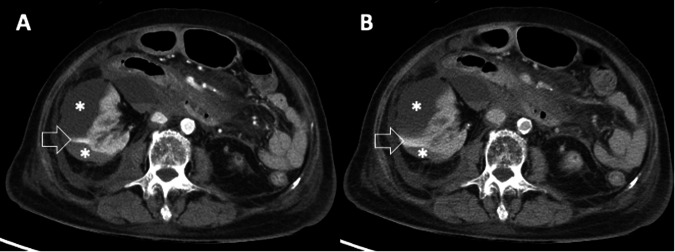
CT images (**A**; arterial phase, **B**; portovenous phase) exemplifying path analysis alignment in a deceased patient during admission. An 80-year-old woman with end-stage renal disease on hemodialysis presented with spontaneous right perinephric hematoma, having prolonged serum partial thromboplastin time (180 s). The CT revealed active vascular contrast extravasation, featuring a minimum length of 11 mm on portovenous phase. She received 4 units of packed red cells within the initial 24 h, was admitted to the intensive care unit for nonsurgical care, and subsequently succumbed after 90 days of admission

In our study, we utilized completed data from 237 out of 272 patients for path analysis, involving 28 estimated parameters. This yielded a ratio of approximately 9 observations per parameter, falling within the suggested range of 5–10 for adequate statistical power and model stability. The aim of this analysis was to dissect the complex interplay among five independent variables (CKD4-5 or dialysis, prolonged PTT, PRC units in 24 h, nonsurgery, and minimum AVCE length > 8 mm) and the dependent variable (ICU admission) in predicting in-hospital mortality. Nonsurgery and ICU admission were considered mediator variables, potentially facilitating the effect of other variables on in-hospital mortality. Following the initial model, five additional models (Fig. E1) were formulated, all demonstrating favorable fit indices (Table E3). Model fit was evaluated using widely recognized indices, indicating excellent fit to the observed data and supporting the adequacy of our sample size. The sixth model (Fig. 3, Table 4, Table E4-E5), characterized by low complexity and significant path coefficients for all included paths, was designated as the final model, indicating robustness in capturing the relationships among the variables of interest. The analysis demonstrated direct influences of CKD4-5 or dialysis (coefficient 0.525, *p* = 0.009), prolonged PTT (coefficient 0.576, *p* < 0.001), and minimum AVCE length ≥ 8 mm (coefficient 0.616, *p* < 0.001) on mortality. The total indirect effect of factors toward mortality was shown with statistical significance (coefficient 0.106, *p* = 0.032), specifically the influences of PRC transfusion via two mediators. A lower rate of PRC transfusion reduced mortality through nonsurgery (coefficient −0.017, *p* = 0.001), whereas a higher rate of PRC transfusion increased mortality through ICU admission (coefficients 0.035, *p* = 0.001), leading to subsequent death (coefficients 0.646–0.749, *p* < 0.001–0.003). Figure 4 illustrates this analysis by presenting a case where multiple factors contributed to in-hospital mortality.

**Table 4:** Univariable relationships of exogenous and endogenous variables and endogenous and endogenous variables used in the path models (*n* = 237)

Variables			Odds ratio (95% CI)	*p*-value
Exogenous		Endogenous		
Packed red cell units in 24 h	→	Nonsurgery	0.902 (0.832, 0.978)	0.012
Prolonged partial thromboplastin time	→	ICU admission	1.804 (1.039, 3.134)	0.036
Packed red cell units in 24 h	→	ICU admission	1.240 (1.118, 1.376)	< 0.001
CKD	→	In-hospital mortality	2.649 (1.258, 5.577)	0.010
Prolonged partial thromboplastin time	→	In-hospital mortality	2.723 (1.523, 4.866)	< 0.001
Packed red cell units in 24 h	→	In-hospital mortality	1.090 (1.012, 1.173)	0.023
Minimum length of AVCE in PVP > 8 mm	→	In-hospital mortality	2.402 (1.236, 4.671)	0.010
**Endogenous**		**Endogenous**		
Nonsurgery	→	ICU admission	0.298 (0.131, 0.677)	0.004
ICU admission	→	In-hospital mortality	3.010 (1.573, 5.757)	< 0.001
Nonsurgery	→	In-hospital mortality	2.731 (1.093, 6.825)	0.032

## Discussion

Our investigation identified factors associated with mortality in patients with abdominopelvic AVCE. Using path analysis, we further explored the complex interrelations among these factors and their effects on in-hospital mortality. The analysis modeled direct and indirect relationships within variables. After model specification, simplification, and evaluation of fit indices, the final model was chosen for its robustness in capturing the relationships among the variables, providing a comprehensive representation of the underlying mechanisms contributing to in-hospital mortality. We identified AVCE size as the only imaging parameter directly impacting mortality, highlighting the critical role of radiologists in not only detecting but also carefully evaluating AVCE features along with the clinical context. Factors such as age, coagulopathy, and vital signs are important considerations for further treatment. Additionally, non-imaging factors such as CKD4-5 or dialysis and prolonged PTT directly impacted mortality, with the latter also indirectly influencing mortality via ICU admission. PRC transfusion affected mortality through nonsurgery and ICU admission. These findings enhance our understanding of complex bleeding-related deaths, which occur through various factors and stages due to complications stemming from the initial blood loss. Some of these factors may be modifiable, offering potential value in clinical practice.

### In-hospital mortality of patients with AVCE

Mortality rates of abdominopelvic AVCE vary according to sites of origin, causes, and underlying conditions. These have been reported to be between 15 and 75% in nontraumatic [8–10] and 18% and 32% in traumatic conditions [6, 11–13]. Our investigation concurred with other reports and showed similar rates of in-hospital mortality in patients with AVCE, which are particularly high for free-space AVCE than the rest.

### Factors associated with in-hospital mortality

Three independent predictive factors (CKD4-5 or dialysis, prolonged PTT, minimum AVCE length) directly influenced in-hospital mortality in our investigation but one (PRC transfusion) acted through mediators. These mediators were nonsurgery and ICU admission.

#### CKD4-5 or dialysis

Our study emphasizes that severe CKD or dialysis independently predicted in-hospital mortality in AVCE patients. Severe CKD can instigate abdominopelvic bleeding, especially within the GI tract [14, 15], and even among those undergoing percutaneous coronary intervention bleeding [16]. Uremic bleeding, observed prior to dialysis, stems from primary hemostasis defects like platelet dysfunction and anemia [17]. Even dialysis patients face persistent bleeding risks due to continuous platelet activation during hemodialysis [18]. Recognizing these underlying conditions is crucial as they can worsen the morbidity and mortality of patients with active bleeding.

#### Prolonged PTT

We found that prolonged PTT, a measure of the intrinsic clotting pathway, played a crucial role in active bleeding scenarios. Prolonged PTT may signify underlying coagulation abnormalities such as liver disease and disseminated intravascular coagulation. Conversely, in cases of ongoing bleeding, prolonged PTT indicates consumptive coagulopathy, where clotting and breakdown occur simultaneously [19]. Previous studies have consistently associated prolonged PTT with heightened morbidity and mortality in bleeding conditions. For instance, in postpartum hemorrhage, individuals with prolonged PTT and low fibrinogen levels were more likely to require surgical intervention and experienced severe outcomes [20]. Similarly, patients receiving heparin with PTT markedly above the therapeutic range faced an increased risk of early mortality [21]. Given the multifaceted nature of active bleeding, our findings underscore the critical role of PTT in predicting in-hospital death.

#### Larger size and progressive heterogeneity of AVCE

The urgency of intervening in AVCE based on CT findings is well-established due to its associated high mortality rates [6]. However, determining which specific characteristics render one AVCE more lethal than another has remained unclear. Our CT analysis identified the minimum AVCE length in the PVP CT as the sole predictor of in-hospital mortality, with a cutoff of 8 mm (AUC 0.589, *p* = 0.017). Additionally, AVCE heterogeneity (measured by SD values) and progression (percentage changes in SD values between CT phases) were correlated with mortality. Heterogeneity suggests a higher bleeding rate, becoming more varied as leaked contrast mingles with blood. While dual- or triple-phase CT scans are typically recommended for suspected nontraumatic hemorrhages [22, 23], our study supports their utility in evaluating patients with suspected AVCE regardless of traumatic or nontraumatic history [24, 25].

#### Treatment other than surgery

Advancements in bleeding control techniques, such as endoscopy and TAE, have reduced the reliance on surgical interventions for active abdominopelvic bleeding. Nontraumatic hemorrhages now favor endoscopic methods for upper and lower GI bleeding [26, 27], while TAE gains prominence for bleeding in solid abdominal organs, the retroperitoneum, and the abdominal wall [8, 10, 28, 29]. Despite these advancements, our study found that surgery yielded better outcomes in terms of in-hospital mortality. Among 43 patients who survived after surgery, 29 had trauma-related active bleeding, predominantly in intramuscular (*n* = 9), intraperitoneal (*n* = 8), and retroperitoneal/extraperitoneal spaces (*n* = 5 each). Surgery’s success may be attributed to specific demographics, with more traumatic cases among survivors, compared to non-survivors with nontraumatic bleeding. This highlights the ongoing significance of surgery in managing abdominopelvic trauma, with procedures like damage-control surgery and exploratory laparotomy playing vital roles [29]. However, potential selection biases may exclude surgically managed patients in poor health, redirecting them to alternative treatments due to perceived risks. This underscores the persistent relevance of surgery as a viable strategy for active bleeding in selected cases, complementing evolving nonsurgical approaches.

#### PRC transfusion

In our study, we found that PRC transfusion indirectly influenced mortality, with lower rates associated with decreased mortality in nonsurgery settings and higher rates linked to higher mortality in ICU settings. The complex interaction of PRC transfusion with other factors preceding deaths was illuminated through our analysis. In cases of hemorrhagic shock, the massive transfusion protocol necessitates large quantities of PRC and fresh frozen plasma in a large quantity [30, 31]. However, a restrictive strategy in maintaining lower hemoglobin levels is increasingly favored in ICU patients with anemia, sepsis, and GI hemorrhage, due to accumulating evidence suggesting mixed or non-beneficial outcomes with PRC transfusion [32, 33]. In trauma cases, the relationship between PRC transfusion and mortality seems to depend on the predicted risk of death, with transfusion associated with increased mortality in patients with a predicted risk below 20% [34]. Our study shed light on the indirect effects of PRC transfusion before death, offering valuable insights for future research.

This investigation has several limitations. First, it is a single-center retrospective study with a relatively small sample size, even though it met the precalculated sample size value. Second, not all instances of AVCE received angiographic, endoscopic or surgical confirmation, potentially affecting the study’s inclusiveness. However, CT reinterpretation by three radiologists served as a reference standard, adhering to established definitions. It is essential to acknowledge that AVCE is an ongoing process influenced by various factors like time, treatment, and underlying pathology, and any visualization method can only provide a single snapshot of the disease’s progression. Third, some factors contributing to mortality may not have been collected and thus were not studied. These may include other blood components such as fresh frozen plasma and pooled thrombocytes. Fourth, consolidating all instances of AVCE, regardless of their underlying causes, into a single category labeled “nontraumatic” might potentially limit the practical applicability of these factors. Nonetheless, this approach has enabled us to gain insights from a broader spectrum of patients with active abdominopelvic bleeding, which was not previously understood. Fifth, in-hospital mortality is significantly confounded by the treatment administered in each case, which can impact the mortality rate. It may not always be secondary to bleeding in the abdomen and pelvis, particularly in trauma patients. In some cases, complications of prolonged hospitalization may have led to death. To address these limitations more effectively, further investigation employing a case-control design or randomized control trial may be beneficial. Finally, we recognize that the near-perfect fit indices of our final path model might raise concerns about overfitting. However, we believe that the model was built on robust theoretical foundations and meticulously specified to distinguish substantive effects from random noise.

In conclusion, several clinical and CT factors had direct and indirect influence on in-hospital mortality in patients presenting with AVCE on abdominopelvic CT. Larger AVCE directly increased mortality, underscoring the crucial role of radiologists in detecting and characterizing these findings.

### Supplementary information

Supplementary information

## Data Availability

The data used to support the findings of this study are included in the article.

## Abbreviations

AP: Arterial phase
AVCE: Active vascular contrast extravasation
CKD: Chronic kidney disease
DP: Delayed phase
PRC: Packed red cell
PTT: Partial thromboplastin time
PVP: Portovenous phase
TAE: Transarterial embolization
