# Supplementary material for: Predictors and pathways of in-hospital mortality in active vascular contrast extravasation detected on abdominopelvic CT
Source: Insights Imaging. 2024 Jul 12;15:174. doi: 10.1186/s13244-024-01748-y (PMC11239638; doi:10.1186/s13244-024-01748-y)
Supplement: Supplementary file 1 — Supplementary information [file 13244_2024_1748_MOESM1_ESM.pdf]

Predictors and pathways of in-hospital mortality in active vascular contrast extravasation detected on abdominopelvic CT

ELECTRONIC SUPPLEMENTARY MATERIAL

Table E1: Definitions and examples of three spaces of active vascular contrast extravasation

Spaces	Definitions	Examples
**Free**	Bleeding could easily spread due to lack of tissues within the space	Lumen of bowel<br>Peritoneal cavity<br>Mesenteric leaves<br>Renal collecting system
**Loose**	Bleeding is less easy to spread due to loosen tissues within the space	Retroperitoneum/extraperitoneum<br>Spleen<br>Subcapsular space of solid organs<br>Subcutaneous tissues<br>Fat-containing solid tumors
**Tight**	Bleeding is least likely to spread, confined within tissues or hematoma	Iliopsoas muscles<br>Rectus sheath or abdominal wall muscles<br>Solid organs except spleen<br>Non-fat-containing solid tumors

Table E2: Univariable analysis of CT findings dependent on in-hospital mortality in patients with active vascular contrast extravasation

Standard deviations of attenuation of AVCE	Unadjusted OR (95% CI)	p-value	Area under the ROC curve (95% CI)	p-value
Delayed phase (HU)	1.034 (1.005, 1.063)	0.019	0.611 (0.522, 0.699)	0.014
Delayed phase >25 HU	8.075 (1.048, 62.240)	0.045		
% Difference between AP and PVP (HU)	1.007 (1.001, 1.013)	0.026	0.612 (0.526, 0.699)	0.011
% Difference between AP and PVP > -20	2.526 (1.205, 5.298)	0.014		
% Difference between AP and delayed phase (HU)	1.012 (0.998, 1.026)	0.089	0.615 (0.516, 0.714)	0.023
% Difference between AP and delayed phase > -55	4.936 (1.100, 22.513	0.037		

AP = arterial phase, AVCE = active vascular contrast extravasation, CI = confidence interval, HU = Hounsfield Unit, OR = odds ratio, PVP = portovenous phase, ROC = receiver operating characteristics

Table E3: Fit indices of the initial and final path models

Model	Chi-square	df	p-value	CFI	TLI	RMSEA	SRMR
Model 1	0.000	0	1.000	1.000	1.000	0.000	0.000
Model 2	0.486	1	0.462	1.000	1.000	0.000	0.011
Model 3	0.574	3	0.902	1.000	1.000	0.000	0.012
Model 4	1.416	5	0.923	1.000	1.000	0.000	0.017
Model 5	5.451	6	0.487	1.000	1.000	0.000	0.030
Final model	2.830	6	0.830	1.000	1.000	0.000	0.023

CFI = comparative fit index, df = degree of freedom, RMSEA = root mean square error of approximation,

SRMR = standardized root mean squared residual, TLI = Tucker-Lewis index

Table E4: Correlation estimates among exogenous variables[1] used in the path models (n=237)

	**Correlation**		**Estimate**
Chronic kidney disease stage 4-5 or dialysis	<—>	Prolonged partial thromboplastin time	0.052
Packed red cell units in 24 hours	<—>	Prolonged partial thromboplastin time	0.051
Minimum length of AVCE in PVP> 8 mm	<—>	Prolonged partial thromboplastin time	-0.158
Packed red cell units in 24 hours	<—>	Chronic kidney disease stage 4-5 or dialysis	0.014
Minimum length of AVCE in PVP> 8 mm	<—>	Chronic kidney disease stage 4-5 or dialysis	0.012
Packed red cell units in 24 hours	<—>	Minimum length of AVCE in PVP> 8 mm	0.250

[1]Exogenous variables were identified from multivariable analysis of predictors of in-hospital mortality in patients with active vascular contrast extravasation.

Table E5: Path coefficients in predicting mortality for patients with active vascular contrast extravasation (n=237)

Path	Standardized coefficient (β)	Standard error (SE)	p-value	Effect type
**Effect on ICU admission**				
Prolonged partial thromboplastin time → ICU admission	0.125	0.062	0.044	Direct
Packed red cell units in 24 hours → ICU admission	0.035	0.011	0.001	Direct
Non-surgery → ICU admission	-0.197	0.092	0.032	Direct
**Effect on non-surgery**				
Packed red cell units in 24 hours → Non-surgery	-0.017	0.005	0.001	Direct
**Effect on in-hospital mortality**				
CKD → In-hospital mortality	0.525	0.201	0.009	Direct
Prolonged partial thromboplastin time → In-hospital mortality	0.576	0.157	<0.001	Direct
Minimum length of AVCE in PVP > 8 mm→ In-hospital mortality	0.616	0.176	<0.001	Direct
Non-surgery → In-hospital mortality	0.749	0.253	0.003	Direct
ICU admission → In-hospital mortality	0.646	0.172	<0.001	Direct
Prolonged partial thromboplastin time → ICU admission → In-hospital mortality (1)	0.081	0.045	0.072	Indirect
Packed red cell units in 24 hours → ICU admission → In-hospital mortality (2)	0.023	0.010	0.022	Indirect

Packed red cell units in 24 hours → Non-surgery → ICU admission → In-hospital mortality (3)	0.002	0.001	0.140	Indirect
Packed red cell units in 24 hours → ICU admission → In-hospital mortality & Packed red cell units in 24 hours → Non-surgery → ICU admission → In-hospital mortality (4)	0.025	0.010	0.016	Indirect
Total indirect effect	0.106	0.049	0.032	Indirect

Figure E1: Initial models of path analysis demonstrate relationships among factors leading to in-hospital mortality in patients with active vascular contrast extravasation detected on abdominopelvic CT. The values were standardized parameter estimates with standard errors in brackets. Values on solid lines indicated statistical significance (p-value < 0.05), while those on dashed lines were not statistically significant. Items in grey boxes directly influenced the outcome (in-hospital mortality), while those in white boxes either indirectly affected (PRC) or acted as mediators (Non-surgery, and ICU) toward the outcome. CKD4-5 = chronic kidney disease stage 4-5, ICU = intensive care unit, PRC = packed red cell, PTT = partial thromboplastin time, PVP = portovenous phase.

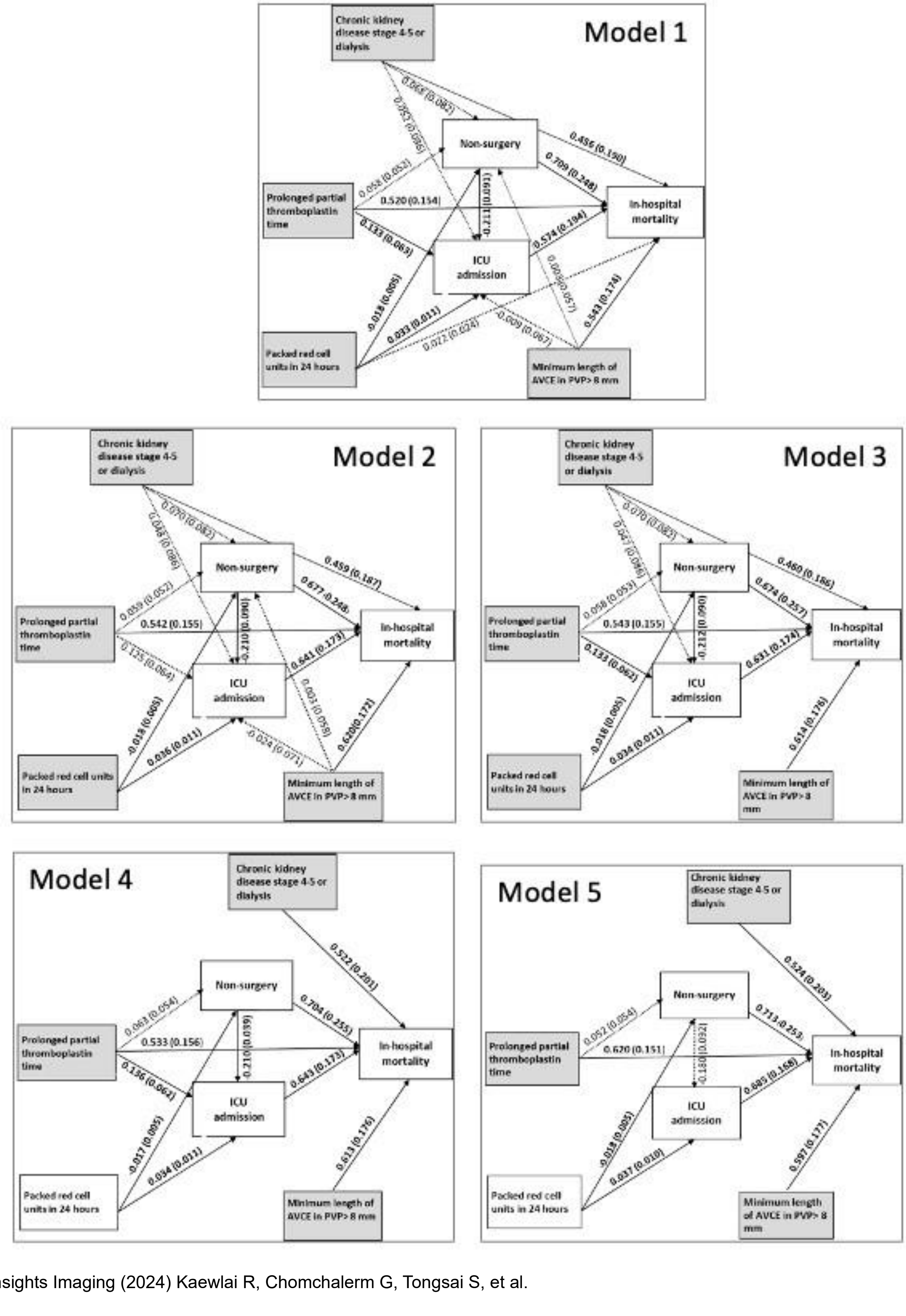
Model 1
Chronic kidney disease stage 4-5 or dialysis
Non-surgery
Prolonged partial thromboplastin time
In-hospital mortality
ICU admission
Packed red cell units in 24 hours
Minimum length of AVCE in PVP> 8 mm
Model 2
Chronic kidney disease stage 4-5 or dialysis
Non-surgery
Prolonged partial thromboplastin time
In-hospital mortality
ICU admission
Packed red cell units in 24 hours
Minimum length of AVCE in PVP> 8 mm
Model 3
Chronic kidney disease stage 4-5 or dialysis
Non-surgery
Prolonged partial thromboplastin time
In-hospital mortality
ICU admission
Packed red cell units in 24 hours
Minimum length of AVCE in PVP> 8 mm
Model 4
Chronic kidney disease stage 4-5 or dialysis
Non-surgery
Prolonged partial thromboplastin time
In-hospital mortality
ICU admission
Packed red cell units in 24 hours
Minimum length of AVCE in PVP> 8 mm
Model 5
Chronic kidney disease stage 4-5 or dialysis
Non-surgery
Prolonged partial thromboplastin time
In-hospital mortality
ICU admission
Packed red cell units in 24 hours
Minimum length of AVCE in PVP> 8 mm
